# Supplementary material for: Mapping the evolution of fertility support policies in China: A content and instrumental analysis
Source: PLoS One. 2025 Oct 9;20(10):e0332137. doi: 10.1371/journal.pone.0332137 (PMC12510515; doi:10.1371/journal.pone.0332137)
Supplement: S1 Appendix — (ZIP) [file pone.0332137.s001.zip › S1 Appendix. 226 original policy documents/64-国务院办公厅转发国家发展改革委关于推动生活性服务业补短板上水平提高人民生活品质若干意见的通知(FBM-CLI-2-5078203).doc]

国务院办公厅转发国家发展改革委关于推动生活性服务业补短板上水平提高人民生活品质若干意见的通知

**发布部门：** [国务院办公厅](https://www.pkulaw.com/law/chl?Aggs.IssueDepartment=601) [机构沿革](javascript:void(0))

**发文字号：**国办函〔2021〕103号

**发布日期：**2021.10.13

**实施日期：**2021.10.13

**时效性：** [现行有效](https://www.pkulaw.com/law/chl?Aggs.TimelinessDic=01)

**效力级别：** [国务院规范性文件](https://www.pkulaw.com/law/chl?Aggs.EffectivenessDic=XC0203)

**法规类别：** [服务](https://www.pkulaw.com/law/chl?Aggs.Category=07102) [营商环境优化](https://dobusiness.pkulaw.com/)

国务院办公厅转发国家发展改革委关于推动生活性服务业补短板上水平提高人民生活品质若干意见的通知
（国办函〔2021〕103号）

各省、自治区、直辖市人民政府，国务院各部委、各直属机构：
　　国家发展改革委《关于推动生活性服务业补短板上水平提高人民生活品质的若干意见》已经国务院同意，现转发给你们，请认真贯彻落实。

国务院办公厅
　　2021年10月13日

关于推动生活性服务业补短板上水平提高人民生活品质的若干意见
（国家发展改革委）

　　近年来，我国生活性服务业蓬勃发展，对优化经济结构、扩大国内需求、促进居民就业、保障改善民生发挥了重要作用，但也存在有效供给不足、便利共享不够、质量标准不高、人才支撑不强、营商环境不优、政策落地不到位等问题。为推动生活性服务业补短板、上水平，提高人民生活品质，更好满足人民群众日益增长的美好生活需要，现提出以下意见。
　　一、加强公益性基础性服务供给
　　**（一）**强化基本公共服务保障。加强城乡教育、公共卫生、基本医疗、文化体育等领域基本公共服务能力建设。认真落实并动态调整国家基本公共服务标准，确保项目全覆盖、质量全达标。以服务半径和服务人口为依据做好基本公共服务设施规划建设。探索建立公共服务短板状况第三方监测评价机制。

　　**（二）**扩大普惠性生活服务供给。在“一老一小”等供需矛盾突出的领域，通过政府购买服务、公建民营、民办公助等方式引入社会力量发展质量有保障、价格可承受的普惠性生活服务。加强普惠性生活服务机构（网点）建设，纳入新建、改建居住区公共服务配套设施规划予以统筹。加强省级统筹，推动县市因地制宜制定社区普惠性生活服务机构（网点）认定支持具体办法，实行统一标识、统一挂牌，开展社会信用承诺。

　　**（三）**大力发展社区便民服务。推动公共服务机构、便民服务设施、商业服务网点辐射所有城乡社区，推进社区物业延伸发展基础性、嵌入式服务。推动大城市加快发展老年助餐、居家照护服务，力争五年内逐步覆盖80%以上社区。支持城市利用社会力量发展托育服务设施。推动构建一刻钟便民生活圈，统筹城市生活服务网点建设改造，扩大网点规模，完善网点布局、业态结构和服务功能。探索社区服务设施“一点多用”，提升一站式便民服务能力。探索建立社区生活服务“好差评”评价机制和质量认证机制。
　　二、加快补齐服务场地设施短板

　　**（四）**推动社区基础服务设施达标。结合推进城镇老旧小区改造和城市居住社区建设补短板，建设社区综合服务设施，统筹设置幼儿园、托育点、养老服务设施、卫生服务中心（站）、微型消防站、体育健身设施、家政服务点、维修点、便利店、菜店、食堂以及公共阅读和双创空间等。开展社区基础服务设施面积条件达标监测评价。

　　**（五）**完善老年人、儿童和残疾人服务设施。推进城乡公共服务设施和公共空间适老化、适儿化改造。在提供数字化智能化服务的同时，保留必要的传统服务方式。建设社区老年教育教学点，推进老年人居家适老化改造。开展儿童友好城市示范，加强校外活动场所、社区儿童之家建设，发展家庭托育点。加快无障碍环境建设和困难重度残疾人家庭无障碍改造，开展康复辅助器具社区租赁。

　　**（六）**强化服务设施建设运营保障。各地补建社区“一老一小”、公共卫生、全民健身等服务设施，可依法依规适当放宽用地和容积率限制。在确保安全规范前提下，提供社区群众急需服务的市场主体可租赁普通住宅设置服务网点。推进存量建筑盘活利用，支持大城市疏解腾退资源优先改造用于社区服务。推广政府无偿或低价提供场地设施、市场主体微利运营模式，降低普惠性生活服务成本。
　　三、加强服务标准品牌质量建设

　　**（七）**加快构建行业性标杆化服务标准。支持以企业为主体、行业组织为依托，在养老、育幼、家政、物业服务等领域开展服务业标准化试点，推出一批标杆化服务标准。加强生活性服务业质量监测评价和通报工作，推广分领域质量认证。推动各地开展生活性服务业“领跑者”企业建设，以养老、育幼、体育、家政、社区服务为重点，培育一批诚信经营、优质服务的示范性企业。

　　**（八）**创建生活性服务业品牌。推动各地在养老、育幼、文化、旅游、体育、家政等领域培育若干特色鲜明的服务品牌。深入实施商标品牌战略，健全以产品、企业、区域品牌为支撑的品牌体系。引导各地多形式多渠道加强优质服务品牌推介。
　　四、强化高质量人力资源支撑

　　**（九）**完善产教融合人才培养模式。支持生活性服务业企业深化产教融合，联合高等学校和职业学校共同开发课程标准、共建共享实习实训基地、联合开展师资培训，符合条件的优先纳入产教融合型企业建设培育库。加快养老、育幼、家政等相关专业紧缺人才培养，允许符合条件的企业在岗职工以工学交替等方式接受高等职业教育。加强本科层次人才培养，支持护理、康复、家政、育幼等相关专业高职毕业生提升学历。到2025年，力争全国护理、康复、家政、育幼等生活性服务业相关专业本科在校生规模比2020年增加10万人。

　　**（十）**开展大规模职业技能培训。强化生活服务技能培训，推进落实农村转移就业劳动力、下岗失业人员和转岗职工、残疾人等重点群体培训补贴政策，对符合条件的人员按规定落实培训期间生活费补贴。在人口大省、大市、大县打造一批高质量劳动力培训基地。逐年扩大生活性服务业职业技能培训规模。

　　**（十一）**畅通从业人员职业发展通道。推动养老、育幼、家政、体育健身企业员工制转型，对符合条件的员工制企业吸纳就业困难人员及高校毕业生就业的，按规定给予社保补贴。做好从业人员职业技能、工作年限与技能人才支持政策和积分落户政策的衔接。关心关爱从业人员，保障合法权益，宣传激励优秀典型。
　　五、推动服务数字化赋能

　　**（十二）**加快线上线下融合发展。加快推动生活服务市场主体特别是小微企业和个体工商户“上云用数赋智”，完善电子商务公共服务体系，引导电子商务平台企业依法依规为市场主体提供信息、营销、配送、供应链等一站式、一体化服务。引导各类市场主体积极拓展在线技能培训、数字健康、数字文化场馆、虚拟景区、虚拟养老院、在线健身、智慧社区等新型服务应用。加强线上线下融合互动，通过预约服务、无接触服务、沉浸式体验等扩大优质服务覆盖面。

　　**（十三）**推动服务数据开放共享。在保障数据安全和保护个人隐私前提下，分领域制定生活服务数据开放共享标准和目录清单，优先推进旅游、体育、家政等领域公共数据开放。面向市场主体和从业人员，分领域探索建设服务质量用户评价分享平台，降低服务供需信息不对称，实现服务精准供给。引导支持各地加强政企合作，建设面向生活性服务业重点应用场景的数字化、智能化基础设施，打造城市社区智慧生活支撑平台。
　　六、培育强大市场激活消费需求

　　**（十四）**因地制宜优化生活性服务业功能布局。推动东部地区积极培育生活性服务业领域新兴产业集群，率先实现品质化多样化升级。支持中西部和东北地区补齐生活服务短板，健全城乡服务对接机制，推进公共教育服务优质均衡发展，完善区域医疗中心布局，加快发展文化、旅游、体育服务。支持欠发达地区和农村地区发展“生态旅游＋”等服务，培育乡村文化产业，提升吸纳脱贫人口特别是易地搬迁群众就业能力。

　　**（十五）**推进服务业态融合创新。在生活性服务业各领域，纵深推动大众创业万众创新。创新医养结合模式，健全医疗与养老机构深度合作、相互延伸机制。促进“体育＋健康”服务发展，构建体医融合的疾病管理和健康服务模式。推进文化、体育、休闲与旅游深度融合，推动红色旅游、工业旅游、乡村旅游、健康旅游等业态高质量发展。促进“服务＋制造”融合创新，加强物联网、人工智能、大数据、虚拟现实等在健康、养老、育幼、文化、旅游、体育等领域应用，发展健康设备、活动装备、健身器材、文创产品、康复辅助器械设计制造，实现服务需求和产品创新相互促进。

　　**（十六）**促进城市生活服务品质提升。开展高品质生活城市建设行动，推动地方人民政府制定生活性服务业发展整体解决方案，支持有条件的城市发起设立美好生活城市联盟。支持大城市建设业态丰富、品牌汇集、环境舒适、特色鲜明、辐射带动能力强的生活性服务业消费集聚区，推动中小城市提高生活服务消费承载力。支持各地推出一批有代表性的服务场景和示范项目，加强城市特色商业街区、旅游休闲街区和商圈建设，集成文化娱乐、旅游休闲、体育健身、餐饮住宿、生活便利服务，打造综合服务载体。

　　**（十七）**激活县乡生活服务消费。加快贯通县乡村三级电子商务服务体系和快递物流配送体系，大力推进电商、快递进农村。建设农村生活服务网络，推进便民服务企业在县城建设服务综合体，在乡镇设置服务门店，在行政村设置服务网点。经常性开展医疗问诊、文化、电影、体育等下乡活动。

　　**（十八）**开展生活服务消费促进行动。推动各地区有针对性地推出一批务实管用的促消费措施，地方人民政府促消费相关投入优先考虑支持群众急需的生活服务领域。深化工会送温暖活动，切实做好职工福利和生活保障，广泛开展职工生活服务项目，为职工提供健康管理、养老育幼、心理疏导、文化体育等专业服务。
　　七、打造市场化法治化国际化营商环境

　　**（十九）**提升政务服务便利化水平。健全卫生健康、养老、育幼、文化、旅游、体育、家政等服务机构设立指引，明确办理环节和时限并向社会公布。简化普惠性生活服务企业审批程序，鼓励连锁化运营，推广实施“一照多址”注册登记。

　　**（二十）**积极有序扩大对外开放。完善外商投资准入前国民待遇加负面清单管理制度。有序推进教育、医疗、文化等领域相关业务开放。支持粤港澳大湾区、海南自由贸易港、自由贸易试验区依法简化审批流程，更大力度吸引和利用外资。探索引入境外家政职业培训机构落户海南。

　　**（二十一）**完善监督检查机制。制定实施重点领域监管清单，梳理现场检查事项并向社会公开，大力推行远程监管、移动监管、预警防控等非现场监管。依托“信用中国”网站、国家企业信用信息公示系统等加强生活性服务业企业信用信息公开，及时公示行政许可、行政处罚、抽查检查结果等信息，加快构建以信用为基础的新型监管机制。

　　**（二十二）**加强权益保障。依法保护各类市场主体产权和合法权益，严格规范公正文明执法。维护公平竞争市场秩序，严厉打击不正当竞争行为。促进平台经济规范健康发展，从严治理滥用垄断地位、价格歧视、贩卖个人信息等违法行为。开展民生领域案件查办“铁拳”行动，从严查处群众反映强烈的预付消费“跑路坑民”、虚假广告宣传、非法集资等案件。
　　八、完善支持政策

　　**（二十三）**加强财税和投资支持。地方各级人民政府要强化投入保障，统筹各类资源支持生活性服务业发展。各地安排的相关资金要优先用于支持普惠性生活服务。落实支持生活性服务业发展的税收政策。发挥中央预算内投资的引导和撬动作用，加强教育、医疗卫生、文化、旅游、社会服务、“一老一小”等设施建设，积极支持城镇老旧小区改造配套公共服务设施建设。对价格普惠且具有一定收益的公共服务设施项目，符合条件的纳入地方政府专项债券支持范围。

　　**（二十四）**加大金融支持力度。积极运用再贷款再贴现等工具支持包括生活性服务业企业在内的涉农领域、小微企业、民营企业发展。引导商业银行扩大信用贷款、增加首贷户，推广“信易贷”，使资金更多流向小微企业、个体工商户。鼓励保险机构开展生活性服务业保险产品和服务创新。

　　**（二十五）**完善价格和用地等支持政策。注重与政府综合投入水平衔接配套，合理制定基础性公共服务价格标准。充分考虑当地群众可承受度以及相关机构运营成本，加强对普惠性生活服务的价格指导。经县级以上地方人民政府批准，对利用存量建筑兴办国家支持产业、行业提供普惠性生活服务的，可享受5年内不改变用地主体和规划条件的过渡期支持政策。对建筑面积300平方米以下或总投资30万元以下的社区服务设施，县级以上地方人民政府可因地制宜优化办理消防验收备案手续。

　　**（二十六）**增强市场主体抗风险能力。健全重大疫情、灾难、事故等应急救助机制，对提供群众急需普惠性生活服务的市场主体特别是小微企业，及时建立绿色通道，强化应急物资供应保障，落实租金减免、运营补贴、税费减免、融资服务等必要帮扶措施。鼓励发展适应疫情常态化防控要求的生活性服务业新业态。
　　九、加强组织实施

　　**（二十七）**健全工作统筹协调机制。国家发展改革委会同有关部门开展高品质生活城市建设行动，各部门按照职责分工组织实施相关建设行动，抓好相关领域和行业支持生活性服务业发展工作，完善行业政策、标准和规范。

　　**（二十八）**压实地方主体责任。县级以上地方人民政府要切实履行主体责任，因地制宜、因城施策编制生活性服务业发展行动方案，研究制定具体措施。省级人民政府要探索将生活性服务业发展纳入市县绩效考核范围，确保各项任务落实落地。

　　**（二十九）**加强统计监测评价。完善生活性服务业统计分类标准，探索逐步建立统计监测制度，建立常态化运行监测机制和多方参与评价机制，逐步形成信息定期发布制度。

　　**（三十）**强化舆论宣传引导。各地区、各部门要广泛宣传动员，加强舆论引导，做好政策解读，主动回应社会关切，及时推广新做法新经验新机制，为生活性服务业发展营造良好氛围。

© 北大法宝：（[www.pkulaw.com](https://www.pkulaw.com/)) 专业提供法律信息、法学知识和法律软件领域各类解决方案。北大法宝为您提供丰富的参考资料，正式引用法规条文时请与标准文本核对。
欢迎查看所有[产品和服务](https://www.pkulaw.com/helps/2.html)。法宝快讯：[如何快速找到您需要的检索结果？](https://www.pkulaw.com/helps/70.html)[法宝](https://www.pkulaw.com/helps/69.html)V6有何新特色？

扫描二维码阅读原文

**原文链接：**<https://www.pkulaw.com/chl/2162c12d4056623ebdfb.html>
